# Attitudes of College Undergraduates Towards Coyotes (*Canis latrans*) in an Urban Landscape: Management and Public Outreach Implications

**DOI:** 10.3390/ani3010001

**Published:** 2013-01-10

**Authors:** Megan M. Draheim, Katheryn W. Patterson, Larry L. Rockwood, Gregory A. Guagnano, E. Christien M. Parsons

**Affiliations:** 1 Virginia Tech Center for Leadership in Global Sustainability, College of Natural Resources & Environment, Virginia Tech, 900 N. Glebe Road, Arlington, VA 22203, USA; 2 Department of Environmental Science and Policy, George Mason University, Fairfax, VA 22030, USA; E-Mails: kpatter3@gmu.edu (K.W.P.); lrockwoo@gmu.edu (L.L.R.); eparson1@gmu.edu (E.C.M.P.); 3 Department of Sociology and Anthropology, George Mason University, Fairfax, VA 22030, USA; E-Mail: gguagnan@gmu.edu

**Keywords:** *Canis latrans*, urban wildlife, attitudes towards wildlife, human-wildlife conflict, human-wildlife interactions

## Abstract

**Simple Summary:**

Understanding the public’s attitudes towards urban wildlife is an important step towards creating management plans, increasing knowledge and awareness about wildlife, and fostering coexistence between people and wildlife. Using undergraduate college students in the Washington, D.C. metropolitan area (where coyotes are a recent arrival), this study examined attitudes towards coyotes and coyote management methods. Amongst other findings, we found differences in opinion between key demographic groups, and respondents grouped management methods into two categories: methods that modified human behavior, and methods that had a direct impact on coyotes. Our results have important implications for coyote management in urban areas.

**Abstract:**

Understanding and assessing the public’s attitudes towards urban wildlife is an important step towards creating management plans, increasing knowledge and awareness, and fostering coexistence between people and wildlife. We conducted a survey of undergraduate college students in the Washington, D.C. metropolitan area—where coyotes are recent arrivals—to determine existing attitudes towards coyotes and coyote management methods. Amongst other findings, we found that the more a person feared coyotes, the less likely they were to support their presence (*p* < 0.001), and the less likely they were to believe that pet owners should be directly responsible for protecting their pets (*p* < 0.001). Respondents demonstrated major gaps in their understanding of basic coyote biology and ecology. Respondents broke wildlife management practices into two categories: those that involved an action on coyotes (both lethal or non-lethal; referred to as “Coyote”), and those that restricted human behavior (referred to as “Human”); the “Human” methods were preferred. We found important differences between key demographic groups in terms of attitudes and management preferences. Our study suggests that wildlife professionals have unique opportunities in urban areas to prevent and reduce conflict before it escalates, in part by targeting tailored outreach messages to various demographic and social groups.

## 1. Introduction

Knowing how to effectively work with humans is as important as knowledge of the natural sciences when dealing with complex wildlife questions [1,2]. Understanding human attitudes towards wildlife issues is an important step in learning how to work with people on these issues—after all, a large part of conservation work is changing human behavior [3] and conservation efforts for predators have been most successful when local attitudes and values have been taken into consideration [4].

Coyotes have naturally expanded their range throughout the continental United States [5]. The mid-Atlantic region was one of the last places colonized by coyotes, and they have only been present in the area for a relatively short time [5], with the first confirmed sighting of a coyote inside Washington, D.C. occurring in 2004 [6]. Although coyotes have not yet become as ubiquitous in this region as in other parts of the country, there have been several human-coyote conflicts in the area, most notably in a sub-division in suburban Maryland [7]. Wildlife managers, non-profit organizations, and others interested in preventing and reducing human-coyote conflict therefore have a unique opportunity in this region to create effective outreach campaigns before conflict escalates. Understanding public attitudes towards coyotes is an important first step in this process.

It is difficult to assess the overall impact of the country’s extensive predator removal system as predator control predates the scientific evaluation of North American ecosystems. However, there is abundant evidence that predator presence in a landscape has profound ecological effects and coyotes have been demonstrated to play an integral part in many ecosystems [8,9,10], including urban and suburban areas. Therefore, given what is known, the ecological impacts from predator control are most likely severe. As such, although it appears that coyotes have survived long-term and extensive lethal control, such programs should still be a cause of concern for conservation biologists. Additionally, some researchers feel that lethal predator control programs might engender negative feelings towards predators in general by the public, which can in turn affect attitudes towards recovery efforts for endangered and threatened predators [11]. 

Predator conservationists have noted that currently the emphasis on predator control is shifting from widespread, non-targeted lethal control to an increased use of non-lethal control methods (targeted at both humans and predators) combined with lethal control targeted to individual animals [12]. Non-lethal methods can be split into two categories: methods that attempt to reduce human-coyote conflict by modifying animal behavior, and methods that attempt to reduce conflict by modifying human behavior [12]. In rural areas, non-lethal coyote control methods address concerns such as livestock protection, and therefore incorporate methods such as fence construction, livestock guarding animals, and disruptive and aversion stimuli on the one hand, and changing livestock husbandry practices on the other. In urban areas, the primary non-lethal method targeted at animal behavior is hazing, but there is no evidence yet that this is effective. Methods targeting human behavior include preventing both intentional and unintentional feeding of coyotes, removing attractants around houses, keeping pets inside at night, and keeping dogs on-leash.

In many cases, non-lethal predator control methods are preferred by the public. For example, Kellert [13] found that while respondents tended to support wolf control to decrease livestock predation, most preferred humane techniques and targeted approaches. Other studies have shown that non-farmers were more likely to prefer non-lethal control methods [14], and that people who live or grew up in urban areas have less support for predator control (especially lethal and non-targeted methods) than those from rural regions [15,16]. 

In many places, urban residents hold positive attitudes towards predators. For example, in Minnesota urban residents held stronger protectionist feelings for and felt more affection towards wolves than rural residents [13]; in Michigan, people who grew up in urban areas had more positive attitudes towards predators than those from rural areas [17]; and, in a meta-analysis looking at studies published between 1972–2000 nation-wide, urban residents consistently had more positive attitudes towards wolves than most rural residents [18]. Traditionally, much of the literature on attitudes towards predators examines the differences between rural and urban residents. As more urban residents are experiencing interactions with wildlife near their homes, this dichotomy might be breaking down.

There are situations where urban residents have negative attitudes towards predators. Heberlein and Ericsson [19] found that multigenerational urban residents (whose parents and perhaps grandparents were also city residents) held more negative views of wolves (and wildlife in general) than those who lived in rural areas or city residents who had regular experiences in rural areas. Furthermore, while most Norwegians favored the existence of larger predators in rural, sparsely populated areas, they had a much lower tolerance when the same species lived closer to urban centers [20]. In the Chicago metropolitan area, coyotes are perceived by residents as being the greatest wildlife-based threat to human health and safety [21], and simply seeing or hearing a coyote can be enough to cause human-coyote conflict; this is true because perception is often a more potent force in establishing attitudes than knowledge [22]. This phenomenon can in part be explained by Western civilization’s tendency to split the world into two binary realms: “culture” (that of the human domain) and “nature” (everything else). This is particularly common in urban areas [23] and has even carried over into conservation biology [24]. In urban and suburban areas, where wildlife is increasingly mingling with large human population centers, the clash between these two realms can cause serious conflict.

While on the one hand, urban residents seem to spend considerable time and expense to attract species such as songbirds, which are viewed positively [25], and indeed report that these interactions provide positive psychological effects [26], they also make considerable efforts to reduce the populations of animals seen as undesirable. Exactly what constitutes an undesirable animal (a “pest”) changes over time and across cultures. For example, Jerolmack [27] traces the rise of the “pest” discourse about urban feral pigeons over time. This discourse did not become dominant until the 1930s, although feral pigeons have lived close to humans for centuries. In many places, coyotes are labeled as pests by some and as valued wildlife by others, causing an ethical and practical challenge for urban wildlife managers and others interested in the subject.

## 2. Methodology and Sample

The goals of the study were three-fold: (1) to begin to understand attitudes towards coyotes in Northern Virginia in an effort to plan for, reduce, and prevent human-coyote conflict; (2) to obtain baseline attitudinal data for the area; and (3) to begin to understand local reactions to various wildlife management techniques.

A survey instrument was developed by the authors, based in part on Jackman’s [28] study of attitudes towards coyotes in Cape Cod. The survey went through a Human Subjects Review Board process and the authors were certified through the same office. 

The survey instrument was administered to undergraduate students at George Mason University (GMU), Fairfax, VA, in September of 2006. GMU is a public institution in the Northern Virginia suburbs of Washington, D.C. that has a diverse student body, closely reflecting the demography of Fairfax County. At the time this data was gathered, 83.4% of the total student body was classified as in-state (GMU Office Student Enrollment census results 2006). While college student attitudes are not necessarily representative of the larger community, they do represent an important demographic in this region. Surveys were administered in introduction to biology and introduction to environmental science courses. These courses meet a general education science requirement, and are therefore open to students in all majors; the majority of students were not science majors. The surveys were distributed to students in laboratory sections at the beginning of the year to ensure that class content was not an influence on their responses, with a response rate of 94.7% (n = 769). 

The questionnaire was comprised of questions that asked participants a range of questions in order to assess their awareness levels of, attitudes towards, knowledge about, and support or fear towards coyotes in the Northern Virginia area. The survey data were coded and entered into an Excel spreadsheet. After checking for coding errors, the data was transferred to SPSS 13 for Mac OS X and analyzed. The survey questions pertaining to respondents’ awareness levels and attitudes about coyotes were assessed based on the responses to knowledge, support, and fear indices as an individual’s knowledge about a species is one of the variables that can affect people’s perceptions of predators [22]. Finally, we create management indices to see how the public perceives management strategies, as human-wildlife conflict management should consider the public’s preferences (among other variables).

### 2.1. Indices

We created four indices (support, fear, coyote, and human) by conducting factor analysis on questions pertaining to each topic using a principal components solution and varimax rotation with Kaiser normalization (according to Kaiser’s rule, factors should not be kept if they explain less of the variation than is contained in a single variable [29]), so that the factors have better predictability. After rotation, only one factor with an eigenvalue of greater than one was retained in the rotated factor matrix, and items possessing factor loadings of 0.40 or greater were interpreted. 

The “support” index measures support for the presence of coyotes in the study area as a means of gathering more information about this aspect of human-coyote interactions. Four items (“To me, coyotes symbolize the beauty and wonder of nature in the D.C. metro area;” “The current D.C. metro area coyote population is a problem;” “Coyotes are a positive addition to our community;” and “Coyotes don’t belong in the D.C. metro area” were used to create this index, based on Jackman [28]. This index was internally reliable (Cronbach’s alpha = 0.643). 

Concerns about living in close proximity to wild animals might help to shape peoples’ attitudes towards these animals [30]. For example, Hook and Robinson [17] found that fear was the factor that most contributed to negative feelings towards predators. Therefore, the “fear” index explored how much respondents feared coyotes (Cronbach’s alpha = 0.868). We also used index items modified by Jackman [28] from Lee and Miller [31] in the creation of the fear index, which included six questions (Table 4). After rotation, only one factor with an eigenvalue of greater than one was retained in the factor matrix, and items possessing factor loadings of 0.45 or greater were interpreted. Independent-samples t-tests were performed to compare gender, age, and whether or not respondents were members of an environmental, wildlife, or animal protection organization with the fear index.

Respondents were also asked to express their preferences for a variety of coyote management techniques. Questions included both lethal and non-lethal techniques. After rotation, three factors were retained; however the third factor was dropped because it did not contain a variable with a factor loading of significance. For the remaining two factors, one was not internally reliable (Cronbach’s alpha = 0.38) and so was discarded. The remaining two sub-indices, titled “Coyote” (Cronbach’s Alpha = 0.77) and “Human,” (Cronbach’s Alpha = 0.71) were internally reliable. 

### 2.2. Pet Ownership

Pet owners are important stakeholders in any discussion of coyote management, as coyotes have preyed on cats and small dogs. Therefore, this project explored the role that pet ownership might have on attitudes towards coyotes. Participants were asked whether they or their household has a dog or cat in the Washington, D.C. metropolitan area. The percentages of dog owners and cat owners were recorded, and a new variable, pet ownership, was created that included both cat and dog owners. Respondents were then asked to rate particular concerns that they might have about dog or cat safety.

Independent-samples *t*-tests were performed that compared whether or not a respondent owned a pet (defined as a dog or cat) with the support and fear indices. Chi-square tests for independence were run to compare pet ownership with support for the existence of coyotes in the D.C. metropolitan area and how much the respondents liked or disliked coyotes. In addition, a Chi-square test for independence was run to compare pet ownership with other questions of interest in the questionnaire.

### 2.3. Demographics

Females made up 63.2% of the subjects while males made up 36.8%. Chi-square tests for independence were run to compare gender with how much support an individual had for the presence of coyotes in the D.C. metropolitan area (Q4) and how much the individual liked or disliked coyotes (Q6).

The ages of the students ranged from 18–47, with a median age of 20 and a mean age of 24. We categorized students as either traditional (18–25; 93.5% of respondents) or non-traditional (26–40; 6.5% of respondents) and then compared their scores on the indices that we created, “Support” and “Fear.” In all cases, there was no statistically significant difference between the scores of the two student categories.

## 3. Results

### 3.1. Awareness and General Attitudes

At the time of the survey, awareness of the presence of coyotes in the Washington, D.C. metropolitan region was fairly low; only 36.7% of respondents (n = 764) were aware that coyotes are present in the area, and only 13.7% (n = 764) remembered seeing or hearing a media report about coyotes in the area in the past year.

Few respondents had extreme opinions when asked how much they did or did not support the existence of coyotes in the study area. Most college students responding to this survey had neutral feelings towards coyotes (Table 1 and Table 2), with 82.3% of respondents either “somewhat” or “not very much” supportive of the existence of coyotes in the study area, and 67.7% feeling neutral about coyotes when asked how much they like or dislike the species.

**Table 1:** Responses to the question: “How much do you support the existence of coyotes in the D.C. metro area?” N = 763. Given in percentages.

	Very much	Somewhat	Not very much	Coyotes should be eliminated or driven out of the D.C. metro area
**Percentage of ** **respondents **	12.2	41.4	40.9	5.5

**Table 2:** Responses to the question: “How much do you like or dislike coyotes?” N = 758. Given in percentages.

	Dislike very much	Dislike somewhat	Neutral	Like somewhat	Like very much
**Percentage of ** **respondents**	4.6	7.5	67.7	13.3	6.9

Respondents were also asked their opinions towards broad coyote management options (Table 3). Most felt that the area’s coyotes should be protected and preserved, but paradoxically at the same time most also felt that the population’s size should be controlled. However, a majority strongly did not support the elimination of the coyote population.

**Table 3:** Attitudes towards coyote management. Given in percentages.

	Strongly agree	Somewhat agree	Neither agree nor disagree	Somewhat disagree	Strongly disagree	N
**The D.C. metro area coyote population should be protected and preserved**	23.8	39.6	26.2	6.8	3.6	692
**The D.C. metro area coyote population size should be controlled**	20.2	45.7	24.1	5.5	4.5	693
**The D.C. metro area coyote population should be completely eliminated**	2.7	5.3	19.0	20.4	52.5	695

### 3.2. Support for Coyotes in the D.C. Metropolitan Area

The support index (Cronbach’s alpha = 0.643) has a minimum possible score of four and a maximum possible score of 20. Most college students responding to this survey had moderate scores on this index (mean = 12.22; median = 12.00; skewness = 0.076, SE = 0.110), with lower scores indicating less support for coyotes.

#### 3.2.1. Fear

There was no clear pattern to the Fear index (Cronbach’s alpha = 0.868) scores (minimum possible and actual score was 6; maximum possible and actual score was 24; mean = 14.37; median = 14.00, skewness = 0.10, SE = 0.09). The responses to individual items in the Fear index provided valuable information by indicating the strongest concerns respondents had about coyotes (Table 4). Of particular interest is that 51.8% of the respondents did not think that a face-to-face encounter with a coyote was a concern, while at the same time 45.5% of respondents felt that the potential for a coyote to attack a child was a major concern. As we will see, this is despite the fact that most respondents knew that coyote attacks on humans are rare (see Knowledge section, below). In addition, over 40% of respondents felt that coyotes spreading rabies was a major concern. The more a person feared coyotes, the less likely they were to support their presence in the area (R = −0.33; *p* < 0.001).

**Table 4:** How concerned respondents are about specific fears related to coyotes. Given in percentages.

	Not a concern	Minor concern	Moderate concern	Major concern	N
**Potential risk to myself in a face-to-face encounter with a coyote**	51.8	21.6	14.9	11.6	726
**Coyotes attacking dogs**	29.6	30.8	26.5	13.1	720
**Coyotes attacking cats**	36.1	27.1	24.4	12.4	726
**Having coyotes near my home**	31.8	26.7	20.1	21.4	738
**Coyotes spreading rabies**	11.3	19.7	26.8	42.2	732
**Coyotes attacking children**	16.1	16.3	22.1	45.5	734

#### 3.2.2. Knowledge

College students responding to this survey were asked a series of true-false questions to determine their level of knowledge about coyotes (Table 5). Of particular interest, 81.2% of respondents agreed that coyote attacks on humans were not common, 77.9% of respondents understood that coyotes will occasionally prey on domestic cats, and 71.2% of respondents knew that if you encounter a coyote, you should not run away from it.

**Table 5:** Responses to knowledge about coyotes questions. The correct response is in bold print and italics for each question. Given in percentages.

	Agree	Disagree	N
Coyotes are carnivores that eat only meat	59.4	**40.6**	769
Coyotes always travel in packs	44.5	**55.5**	762
Coyote attacks on humans are not common	**81.2**	18.8	759
Adult male coyotes weigh on average 100 lbs.	57.8	**42.2**	761
Coyotes are in danger of becoming extinct	70.2	**29.8**	762
Coyotes will kill cats on occasion	**77.9**	22.1	760
If you encounter a coyote, you should run away from it	28.8	**71.2**	761

On the other hand, respondents lacked knowledge of basic coyote biology and ecology. For example, 59.4% thought that coyotes were obligate carnivores with no vegetation in their diet (in fact, vegetation can be an important component of coyote diets [32,33,34]), 57.8% of respondents believed that the average weight of an adult male coyote was 100 pounds (Nowak [35] reports coyote weights between about 18 to 44 pounds, while the larger Northeastern coyotes can be larger), and 70.2% of respondents believed that coyotes were in danger of becoming extinct, while this is clearly not the case. This is similar to the findings of Kellert and Berry’s [36] study, where about 60% of respondents believed the statement: “Timber wolves, bald eagles, and coyotes are all endangered species of animals.” 

#### 3.2.3. Pet Ownership

Pet ownership can play a role in influencing attitudes towards other animals. For example, pet ownership has been correlated with more favorable attitudes towards urban wildlife than non-pet owners [37], and having a positive experience with a pet can increase positive attitudes towards other animals as well [38]. Conversely, an attachment to animals (in this case, sheep) has been linked to negative feelings towards large carnivores in rural Norway [39]. The fact that coyotes have been known to prey on pets (including cats and small dogs) could therefore play a role in pet owners’ attitudes towards the species.

The majority of respondents (56.9%; n = 431) were not pet owners (defined here as owning at least one dog and/or cat), while 43.1% (n = 326) had one or more pets. Of those, 32.7% owned at least one dog, and 19.7% owned at least one cat.

Most respondents, whether pet owners or non-pet owners, were either not concerned or had only minor concerns about dogs being attacked by coyotes. Pet owners were most concerned about dogs being hit by vehicles or fighting with other dogs, while non-pet owners were concerned about vehicles and dogs being stolen for dog fighting (Table 6 and Table 7). 

**Table 6:** Pet owners’ responses to: “Please indicate how concerned you are that the following could happen to dogs that are outside unsupervised or are off-leash in the D.C. metro area.” Given in percentages.

	Not a concern	Minor concern	Moderate concern	Major concern	N
**Fighting with other dogs**	15.9	33.0	33.3	17.8	315
**Being hit by a car or truck**	5.6	14.9	27.6	51.9	322
**Being attacked by a coyote**	38.9	38.5	13.1	9.6	314
**Being stolen for dog fighting**	44.2	28.2	14.4	13.1	312

**Table 7:** Non-pet owners’ responses to: “Please indicate how concerned you are that the following could happen to dogs that are unsupervised and off-leash in the D.C. metro area.” Given in percentages.

	Not a concern	Minor concern	Moderate concern	Major concern	N
**Fighting with other dogs**	22.9	29.9	34.6	12.7	402
**Being hit by a car or truck**	12.5	16.1	33.7	37.7	409
**Being attacked by a coyote**	34.5	27.2	19.6	18.6	397
**Being stolen for dog fighting**	37.8	21.1	20.6	20.6	389

Even fewer respondents (both pet owners and non-pet owners) were concerned about coyotes attacking cats. Again, being hit by a car or truck was seen as the greatest threat for both pet owners and non-pet owners, while being attacked by dogs was seen as a moderate or major concern by many. Perhaps surprisingly, non-pet owners were more concerned about cats being attacked by coyotes than pet owners (Table 8 and Table 9).

**Table 8:** Pet owners’ responses to: “Please indicate how concerned you are that the following could happen to cats that are unsupervised and off-leash in the D.C. metro area.” Given in percentages.

	Not a concern	Minor concern	Moderate concern	Major concern	N
**Being attacked by dogs**	28.1	37.1	19.7	15.2	310
**Being hit by a car or truck**	17.4	20.6	27.1	34.8	310
**Being attacked by a coyote**	45.7	31.9	12.2	10.2	304
**Being attacked by great horned owls or hawks**	51.7	28.1	10.6	9.6	302

**Table 9:** Non-pet owners’ responses to: “Please indicate how concerned you are that the following could happen to cats that are unsupervised and off-leash in the D.C. metro area.” Given in percentages.

	Not a concern	Minor concern	Moderate concern	Major concern	N
**Being attacked by dogs**	32.8	28.9	25.2	13.0	408
**Being hit by a car or truck**	20.7	21.9	29.9	27.5	411
**Being attacked by a coyote**	42.3	24.7	18.9	14.1	397
**Being attacked by great horned owls or hawks**	47.7	24.7	15.8	11.7	392

Non-pet owners feared coyotes more than pet owners (t_(664)_ = 3.00; *p* < 0.01). Although there was no significant difference between pet owners and non-pet owners in their levels of the “Support” index discussed in the methods, a chi-square test for independence comparing pet ownership to support for the presence of coyotes in the region demonstrated that more pet owners supported the existence of coyotes in the study area than non-pet owners (Pearson Chi-Square = 8.24, Cramer’s V = 0.10, *p* = 0.041; see Table 10).

**Table 10:** “How much do you or don’t you support the coyote’s existence in the D.C. metro area?”.

	Pet owners	Non-pet owners
**Very much**	15.4%	9.7%
**Somewhat**	43.1%	40.1%
**Not very much**	36.3%	44.3%
**Coyotes should be eliminated or driven out of the D.C. metro area**	5.2%	5.8%
**Totals**	100%	99.9%

A chi-square test for independence also compared pet ownership with how much respondents liked or disliked coyotes (Table 11). Pet owners seemed to have more extreme feelings towards coyotes, either positively or negatively, than non-pet owners, which is consistent with what Bjerke *et al.* (2003) reported [37]. (Pearson Chi-Square = 11.88, Cramer’s V = 0.13, *p* = 0.018).

**Table 11:** “How much do you like or dislike coyotes?” Results of a Chi-square test for independence.

	Pet owners	Non-pet owners
**Dislike very much**	5.2%	4.2%
**Dislike somewhat**	5.2%	8.9%
**Neutral**	65.2%	69.6%
**Like somewhat**	14.5%	12.6%
**Like very much**	9.8%	4.7%
**Total**	99.9%	100.0%

The majority of students in our sample (93%) had no direct knowledge of either a dog or a cat being attacked by a coyote. Of the remaining 7%, some attacks had been witnessed or respondents felt that there was other evidence that pointed to a coyote’s involvement, but in many cases respondents assumed that a coyote was the cause for the disappearance of a pet although there was no direct evidence. However, for the purposes of this study, the belief that a coyote was involved was as important as whether a coyote was actually involved, because perception can play a role in establishing attitudes. Therefore, we were able to include all instances where a pet was reportedly killed by a coyote, whether or not there was evidence to support this claim [22]. Although knowing about an attack on a pet did not affect respondents “Fear” or “Support” scores, it did seem to have an impact on how much a person liked or disliked coyotes. Fewer respondents who knew of a pet attack felt neutrally towards coyotes, and more people who had knowledge of such an attack disliked coyotes than those who did not know of an attack (Pearson Chi-Square = 21.235, Cramer’s V = 0.20, *p* < 0.001; see Table 12).

**Table 12:** The relationship between knowledge of a pet attack by a coyote and how much the respondents liked or disliked coyotes. Results of a chi-square test for independence.

	Have knowledge of a pet attack	Have no knowledge of a pet attack
**Dislike very much**	15.1%	3.9%
**Dislike somewhat**	5.7%	7.6%
**Neutral**	54.7%	68.6%
**Like somewhat**	9.4%	13.7%
**Like very much**	15.1%	6.3%
**Total**	100.0%	100.1%

Respondents were asked how much they agreed or disagreed with the statement: “If people allow their pets outside unsupervised, they should not blame coyotes for pets that are attacked.” Most respondents agreed or felt neutrally about this statement (Table 13). A chi-square test for independence compared the relationship between pet ownership and this statement, but no significant difference between the two groups was found. Both pet owners and non-pet owners seem to agree that pet owners have some responsibility to keep their pets safe from coyotes.

**Table 13:** Level of agreement with whether coyotes should be blamed for predations of unsupervised pets. Given in percentages.

	Strongly agree	Somewhat agree	Neither agree nor disagree	Somewhat disagree	Strongly disagree	N
**Q26. If people allow their pets outside unsupervised, they should not blame coyotes for pets that are attacked**	19.7	34.3	19.2	16.0	10.8	720

The “Fear” index and responses to the above statement were negatively correlated (Pearson correlation coefficient = −0.20, *p* < 0.001) so that the less a person feared coyotes, the more he/she was likely to agree that people should not blame coyotes for attacks if pets are left outside unsupervised. The “Support” index and responses to the above statement were positively correlated (Pearson correlation coefficient = 0.25, *p* < 0.001), so that the more a person agreed with the statement, the more support for the existence of coyotes in the study area he/she was likely to have.

#### 3.2.4. Wildlife Management

While previous research has explored the preference between lethal and non-lethal management methods [13,14,15,16], the present factor analysis pointed to slightly different categories (Table 14). The “Coyote” index (Cronbach’s Alpha = 0.77) included both the lethal and one non-lethal technique (“Implement birth control or sterilization measures to keep coyote populations in check”); all required an action being taken on individual coyotes. The “Human” index (Cronbach’s Alpha = 0.71) included methods that restricted human activities in some manner in order to reduce human-coyote conflict. Respondents tended to prefer the “Human” management techniques (minimum possible score = 1, maximum possible score = 5; mean = 3.98; skewness value < 0.001; Kurtosis value < 0.001) to the “Coyote” management techniques (minimum possible score = 1, maximum possible score = 5; mean = 2.37; skewness value < 0.001; Kurtosis value = 0.001). 

**Table 14:** Wildlife Management Indices.

Coyote (Cronbach’s alpha = 0.77)	Human (Cronbach’s alpha = 0.71)
Kill as many coyotes as possible Kill specific coyotes that attack humans Kill specific coyotes that attack pets Implement birth control or sterilization measures to keep coyote populations in check	Preserve natural areas to serve as a buffer between humans and coyotes Warn residents to keep cats inside and dogs on leash with supervision Warn residents to remove food sources from outside Prohibit the intentional feeding of coyotes

#### 3.2.5. Gender

Many studies have examined the role that gender plays in predicting attitudes [16,40,41]. We examined the effect gender had on the outcome of several of the survey items. Based on a chi-square test for independence, men tended to have more of an affinity towards coyotes more than women (*p* = 0.002, Pearson Chi-Square = 17.449, Cramer’s V = 0.20; Table 15). Similarly, men tended to support the presence of coyotes in the study area more than women (*p* = 0.007, Pearson Chi-Square = 12.076, Cramer’s V = 0.13; Table 16).

**Table 15:** The relationship between gender and how much the respondents liked or disliked coyotes. Results of a Chi-square test for independence.

	Female	Male
**Dislike very much**	5.7%	2.9%
**Dislike somewhat**	9.3%	4.3%
**Neutral**	68.6%	66.3%
**Like somewhat**	10.9%	17.4%
**Like very much**	5.5%	9.1%
**Total**	100.0%	100.0%

**Table 16:** The relationship between gender and how much the respondents supported the presence of coyotes in the D.C. metro area. Results of a Chi-Square test for independence.

	Female	Male
**Very much**	9.6%	16.5%
**Somewhat**	39.6%	43.5%
**Not very much**	44.4%	35.6%
**Coyotes should be eliminated from the D.C. metro area**	6.3%	4.3%
**Totals**	99.9%	99.9%

Women had higher scores on the “Fear” index than men (t_(580)_ = 5.33, *p* < 0.01), and men had stronger “Support” scores than women, demonstrating again that men had more support for the presence of coyotes in the study site (t_(488)_ = −3.67, *p* < 0.01). 

Men tended to prefer the management actions listed in the “Coyote” index (t_(741)_ = −2.83, *p* < 0.01), while women preferred the management methods in the “Human” index (t_(746)_ = 5.47, *p* < 0.01). 

## 4. Discussion

Awareness of coyote presence tended to be low in the study sample population. Perhaps because of this, most respondents had neutral feelings towards coyotes and some level of support for their presence in the study area. Individuals who hold non-extreme attitudes about a species might be more open to persuasive arguments than those who fall further along the attitudinal spectrum, either positively or negatively [42]. As negative feelings can be engendered through negative encounters with animals [37], wildlife managers may want to reach local residents before conflict escalates with proactive messages about the steps to take to reduce and prevent human-coyote conflict. As coyotes are an emerging species in the study area, follow-up studies that track if and how attitudes change as awareness grows would be useful.

The more a person feared coyotes, the less likely they were to tolerate the presence of coyotes in the study area. Public outreach and educational approaches about coyotes are already part of most management plans, and wildlife managers should be encouraged to increase their efforts to both present the public with materials about living with coyotes, and to take steps to address and ease the public’s concerns as a management strategy. Although most respondents understood that coyote attacks on people were rare, and most were not concerned with the potential risk to themselves from an encounter with a coyote, the potential risk to a child from a coyote attack was one of the strongest concerns that respondents had about living near coyotes. These findings suggest that outreach programs might best tailor their messages to the public to address specific concerns. For example, Vancouver’s highly successful coexistence with coyotes program spends a portion of their time and resources teaching children what to do when they see a coyote (e.g., wave their arms around to appear big, yell at the coyote, throw objects near the coyote, and do not run). Other program components have focused on stopping intentional and unintentional feeding of coyotes, in recognition that food conditioning is a major contributor to human-coyote conflict [43]. 

Most of the sample population demonstrated some basic knowledge of coyotes and of what it means to live in an area with resident coyotes. For example, they understood that they should not run away from a coyote if one is encountered, and that coyotes might prey on cats. Knowing this might make people more likely to take proactive steps to protect their pets (by not leaving them outside unattended from dusk to dawn, not leaving pet food outside, *etc.*) which could help to prevent conflict and perhaps increase tolerance for coyotes by preparing people for direct and indirect encounters. 

On the other hand, respondents also had some basic misunderstandings about coyotes. Most believed that male coyotes weighed an average of 100 pounds, although coyotes are smaller than this. It is possible that larger animals engender more fear, and in fact anecdotally the authors have noticed that when members of the public learn the actual size of coyotes, fear levels appear to decrease, at least in the short-term. This might help to defuse potential conflict situations and allow wildlife managers to come up with solutions that are beneficial to both residents and the local coyote population. 

Most respondents, whether or not they owned a pet, were not very concerned with the potential for a dog or cat to be attacked by a coyote (the threat of moving vehicles was seen as being a much greater threat). In addition, the majority of both pet owners and non-pet owners felt that coyotes should not be blamed for pet predations when the dog or cat was left outside unsupervised, an encouraging finding that could be built on with outreach materials that stress the importance of pet owner responsibility. Perhaps not surprisingly, the more people feared and/or disliked coyotes, the less likely they were to agree that pet owners were responsible for such events. 

Although there were no differences in the average “Support” scores for pet owners and non-pet owners, those without pets were more likely to be afraid of coyotes. Overall, pet owners seemed to have more extreme attitudes towards coyotes, either negatively or positively, than non-pet owners, perhaps because some pet owners were more prone to like all animals, while others were more concerned about potential threats to their pets. As a result, it might be useful to target pet owners as a specific group in outreach campaigns in order to develop management strategies that the public will both respond to and agree with. Understanding the reasons why pet owners seem to have more extreme attitudes is an important avenue for future research. 

Although we hypothesized that wildlife management preferences would fall into a lethal/non-lethal division, this was not the case. Rather, respondents tended to group management techniques that involve doing something to coyotes—lethal or non-lethal—together, while grouping methods that involved restricting human activities in another category. These methods included prohibitions (about feeding coyotes), issuing warnings (to keep pets inside or on leash and not to feed pets outside), and preservation policies (protect natural areas). The methods directed at humans proved to be favored over those directed at coyotes. Wieczorek Hudenko *et al.* [44] had a related finding: more than half of their respondents believed that human-coyote interactions could be reduced through human behavior change. This belief has been borne out in other studies as many coyote attacks appear to be related to coyotes that have been habituated to humans or food conditioned through direct or indirect feeding [45]. When creating coyote management plans, wildlife managers should take into account the fact that the public might not see methods as either lethal or non-lethal, and that in urban areas residents might oppose any action taken on coyotes.

Gender played a predictive role in determining attitudes both towards coyotes and towards wildlife management preferences. While men tended to like coyotes more, support their presence in the area more, and fear them less, women perhaps paradoxically preferred the “Human” management techniques that did not directly impact coyotes. Other studies have found that men are more likely to support the use of lethal coyote control methods than women [16,41]. Gender should be taken into consideration when preparing outreach programs.

Personal experience can play a key role in forming attitudes, both negatively and positively [37]. For example, one study found that attitudes towards species are directly related to whether or not that species causes an individual harm or inconvenience, as defined by the individual [20]. Another study found that a negative experience with an urban species increased negative feelings towards all species that are commonly thought to come into conflict with humans in urban areas [37]. In areas only recently populated by coyotes, wildlife managers and outreach groups can play an important role by decreasing the chances of conflict and therefore decreasing the chances that negative attitudes towards coyotes will become deeply entrenched.

Attitudes, especially strong attitudes, might be formed mainly by early, formative experiences [46]. However, educational efforts can still play a vital role when targeting those who feel neutral or ambivalent about animals, as there is some evidence to suggest that persuasive arguments can influence those with weakly held beliefs and attitudes [42]. As the majority of respondents seemed to hold neutral attitudes towards coyotes, wildlife managers and others interested in human-coyote coexistence potentially have an opportunity to make an impact in urban areas where coyotes have been present for only a short time.

## 5. Conclusions

In conclusion, we consider this survey to be the first step towards a broader understanding of attitudes towards coyotes in this region. Because of its limited sample, readers should be hesitant to extrapolate our results to the population as a whole. As such, further research should explore attitudes and knowledge in other local populations.
